# Three-Dimensional Imaging Based on Refractive Camera Model and Error Calibration for Risley-Prism Imaging System

**DOI:** 10.3390/s26072013

**Published:** 2026-03-24

**Authors:** Wenjie Luo, Shumin Yang, Duanhao Huang, Feng Huang, Pengfei Wang

**Affiliations:** School of Mechanical Engineering and Automation, Fuzhou University, Fuzhou 350108, China; 240220080@fzu.edu.cn (W.L.); y18965808901@163.com (S.Y.); 230210002@fzu.edu.cn (D.H.); huangf@fzu.edu.cn (F.H.)

**Keywords:** Risley-prism, 3D reconstruction, error analysis, error calibration

## Abstract

Three-dimensional (3D) reconstruction technology has found widespread applications across various domains, including intelligent driving and underwater exploration. But the existing imaging systems and methods still have deficiencies in terms of reconstruction accuracy, detection distance and system volume. Herein, this paper presents a three-dimensional detection and reconstruction method based on a compact Risley-prism 3D imaging system that achieves multi-viewpoint imaging by rotating the Risley prism to adjust the camera’s optical axis. A refractive camera model that integrates the pinhole camera model with the vector form of Snell’s law is established to precisely describe beam trajectory. A forward projection method suitable for refractive interfaces is developed based on Fermat’s principle, and the influence of systematic errors on the reconstruction is analyzed in detail through simulation. Furthermore, a new 3D reconstruction method combining error calibration based on the optimization iteration is introduced to avoid the influence of error and improve reconstruction quality. Experimental results demonstrate that the proposed approach markedly enhances 3D reconstruction accuracy, reducing the Normalized Root Mean Square Error (NRMSE) from 0.9076 to 0.0207.

## 1. Introduction

For humans, the acquisition of external information predominantly relies on the integration of multiple sensory modalities; visual perception serves as the primary channel and can obtain information accounting for 80% of the total information [1]. Traditional visual information processing has largely been confined to two-dimensional (2D) images. However, driven by the rapid advancements in computer vision technology, three-dimensional (3D) imaging, which provides a more comprehensive framework for information representation, has garnered increasing attention from both the academic and industrial communities. So far, 3D reconstruction technology has demonstrated significant potential and practical utility in emerging application domains such as autonomous driving, underwater exploration and 3D metrology [2,3,4].

The 3D reconstruction technology can be classified into two major categories based on different data acquisition methods: active 3D reconstruction and passive 3D reconstruction. Active 3D reconstruction technology acquires three-dimensional information of objects by actively projecting light, including the structured light method [5,6], time-of-flight method [7,8] and triangulation method [9]. Compared with active 3D reconstruction technology, passive 3D reconstruction relies on natural light in the external environment or existing image data to restore 3D information, including binocular stereo vision [10,11], structure from motion [12,13] and other technologies, which have been widely applied in large-scale scene modeling and environmental perception. However, the above-mentioned methods have inherent flaws and limitations. Active 3D reconstruction requires light sources or signal transmitters, and the equipment is often relatively expensive. Currently, passive 3D reconstruction requires multiple cameras or a large spatial layout, which is difficult to apply in some scenarios with limited space.

In response to the above problems, using monocular cameras to achieve 3D reconstruction has become an important and feasible solution. At present, there are already some 3D reconstruction methods based on single views, which usually rely on prior knowledge, such as the geometric relationships of objects in the image, for reconstruction. However, in most practical applications, due to the lack of geometric constraint information between target objects, the restoration process of three-dimensional structures becomes rather complex, and there are still bottlenecks in terms of reconstruction accuracy and overall performance. To enhance the accuracy of 3D reconstruction, obtaining multi-view image information is of vital importance. Therefore, how to obtain multi-view images using a single camera is the key.

To overcome this challenge, research has shown that the introduction of specific optical components into the camera’s optical path can effectively improve system performance [14]. Based on differences in optical principles, optional optical elements can primarily be categorized into three types: mirror reflection, grating diffraction, and prism refraction [15,16,17,18]. The 3D imaging system based on the Risley prism is a typical representative of refractive systems. Compared to existing 3D imaging systems, the Risley-prism 3D imaging system features a more compact structure, less space, reduced sensitivity to environmental disturbances and higher system integration [19,20], making it suitable for space-constrained applications such as portable 3D scanners or underwater imaging platforms. Unlike mirror-based or grating-based systems, Risley prisms offer a refractive mechanism for beam steering, which avoids occlusion and maintains a stable optical path length, thereby simplifying system calibration and enhancing robustness in dynamic environments. Additionally, the Risley-prism 3D imaging system has become a hot topic of research due to its precise control of the camera’s visual axis through the rotational movement of the double prism, enabling the acquisition of muti-angle images while keeping the camera stationary.

The Risley-prism 3D imaging system is designed to perform high-precision 3D reconstruction using a compact and stationary monocular camera. Its primary function is to achieve multi-viewpoint imaging by independently rotating a pair of prisms to deflect the optical axis. Key technical indicators for such a system include reconstruction accuracy (e.g., Normalized Root Mean Square Error—NRMSE), detection range, system volume, and robustness to systematic errors. Existing systems often fall short in balancing these indicators, particularly in achieving both high accuracy and compactness, which motivates the development of a more accurate refractive camera model and a dedicated error calibration framework.

To date, much research has been carried out utilizing Risley-prism imaging systems to realize 3D detection. Liu et al. [21] proposed an image distortion correction and 3D reconstruction method by establishing an equivalent virtual camera model. Zhang et al. [22] utilized homography matrices to obtain image arrays and achieved multi-view 3D reconstruction, but significant errors were still observed in the reconstruction of distant targets. In addition, more recent advances in 3D reconstruction methods and imaging approaches, such as those applied to concrete dam structural health monitoring [23], architectural structure reconstruction [24], defect detection in urban underground pipeline scenes [25], and tiny crack measurement using parallel laser line-camera systems [26], have demonstrated the growing demand for high-accuracy, compact 3D imaging solutions. However, these methods typically rely on conventional camera configurations or specialized scanning mechanisms that may not offer the same combination of compactness and flexibility as Risley-prism systems.

Despite these advantages and studies, several technical bottlenecks remain. Accurately modeling the non-linear refraction introduced by the prisms is non-trivial. The conventional pinhole camera model is inadequate, necessitating the development of a specialized refractive camera model. Furthermore, system errors—such as manufacturing inaccuracies and mechanical misalignments during installation—significantly degrade reconstruction quality. While previous studies have explored error analysis for Risley-prism systems in beam steering applications [27,28,29,30], a comprehensive investigation focused on their impact on 3D reconstruction accuracy is still lacking.

To address these specific challenges, this paper makes the following contributions:

- A refined refractive camera model is proposed by integrating the pinhole camera model with the vector form of Snell’s law, theoretically eliminating inherent model inaccuracies.
- A forward projection method based on Fermat’s principle is developed to simulate the imaging process systematically.
- A detailed simulation analysis quantifies the impact of various system errors on 3D reconstruction accuracy.
- A novel 3D reconstruction method incorporating error calibration via optimization iteration is introduced, effectively mitigating error influences and significantly enhancing reconstruction quality.

## 2. Ray Tracing and 3D Reconstruction Methods for the Risley-Prism System

### 2.1. System Structure and Principle

**Figure 1:**
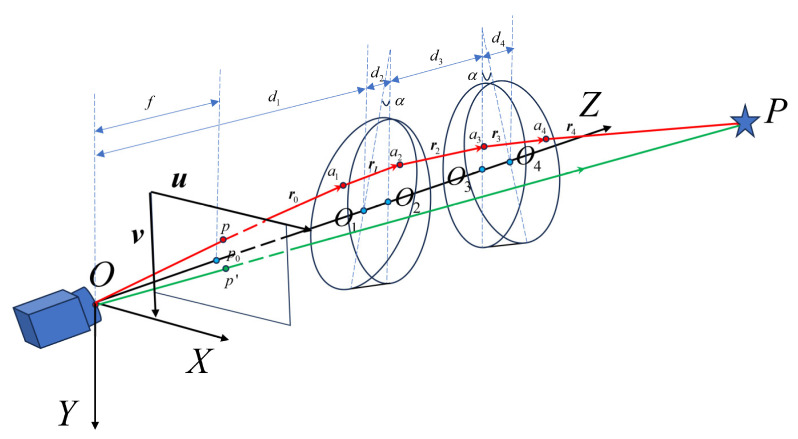
Schematic diagram of the Risley-prism 3D imaging system.

The Risley-prism 3D imaging system mainly consists of a fixed camera and a pair of rotating prisms that can be independently rotated around the *Z*-axis, as shown in Figure 1. The prism that is closer to the camera is defined as Prism 1, and the one that is farther from the camera is defined as Prism 2. The rotation angle is zero when the main cross section of the prisms lies in the *YOZ* plane, with the thick end pointing in the positive direction of the Y-axis. The angle at which the prism rotates clockwise is defined as a positive value. The rotation angles of Prism 1 and Prism 2 are denoted as $\theta_{1}$ and $\theta_{2}$ respectively. The two prisms usually use the same design parameters, with wedge angles of α, refractive indices of n, and the refractive index of air as $n_{0}$. Point $O_{i+1}$ represents the intersection of the Z-axis and the prism plane, i=0,1,2,3. The distance from $O_{1}$ to the optical center of the camera O is $d_{1}$, while $d_{2}$ and $d_{4}$ represent the thickness of Prism 1 and Prism 2 along the Z-axis, respectively. The distance between the two prisms’ perpendicular planes is denoted as $d_{3}$. By rotating the prisms to control the refraction path of the incident light beam, the system can achieve multi-viewpoint imaging without changing the camera position.

The Risley-prism assembly is conventionally recognized for its capability in precise two-dimensional beam steering. However, its application can be effectively extended to 3D imaging by leveraging its beam deflection principle to emulate a multi-view imaging system. The key lies in utilizing the independent rotations of the prisms to project the camera’s optical axis onto diverse spatial directions, thereby capturing a set of images of the static scene from multiple viewpoints while the camera remains physically stationary. This collection of multi-perspective images provides the essential parallax information required for 3D reconstruction.

### 2.2. Ray Tracing Method Based on the Refractive Camera Model

For the common imaging system, the imaging process is typically modeled using the pinhole camera model based on linear perspective projection. However, due to the introduction of Risley prisms, light rays are refracted as they pass through the prisms, resulting in significant nonlinear optical characteristics. Consequently, the pinhole camera model is not suitable for 3D reconstruction tasks in this system. To address this critical problem, the refractive camera model is utilized to accurately model the system and accommodate its complex nonlinear characteristics. By combining the pinhole camera model with the vector form of Snell’s law, a new imaging model that allows for individual ray tracing for each pixel in the image to determine the position of an object point in real space is established.

Let $P={\left(X_{p},Y_{p},Z_{p}\right)}^{T}$ be an arbitrary object point in space. In the absence of Risley prisms in front of the camera, the ideal pixel coordinates on the image plane would be $p^{\prime }={\left[u^{\prime },v^{\prime }\right]}^{T}$. According to the pinhole camera model, the relationship between the object point P and the ideal image point $p^{\prime }$ can be described by the following equation:

(1) $$\lambda {\bar{p}}^{\prime }=K\left[R\;|\;T\right]P$$

(2) $$K=\left[\begin{array}{ccc} \frac{f}{dx} & 0 & u_{0}\\ 0 & \frac{f}{dy} & v_{0}\\ 0 & 0 & 1 \end{array}\right]$$

where λ is the scale factor and ${\bar{p}}^{\prime }$ represents the homogeneous coordinates of $p^{\prime }$. The matrix R is a 3 × 3 rotation matrix, and T is a 3 × 1 translation vector. The matrix K represents the intrinsic parameters of the camera; f denotes the focal length of the camera; dx and dy are the pixel dimensions in the *X* and *Y* directions, respectively, and $\left(u_{0},v_{0}\right)$ are the coordinates of the principal point $p_{0}$, which is the intersection of the optical axis of the camera and the image plane.

The distortion can be introduced by the camera lens and primarily arises from radial distortion [31]. Radial distortion is closely related to the distance between the ideal image point and the principal point. The relationship between the distorted image point $p_{d}^{\prime }$ and the ideal image point $p^{\prime }$ is as follows:

(3) $$p_{d}^{\prime }=\left(\begin{array}{c} 1+k_{1}r^{2}+k_{2}r^{4}+k_{3}r^{6} \end{array}\right)p^{\prime }$$

where $k_{1},k_{2},k_{3}$ are the radial distortion coefficients and r is the distance from the ideal image point $p^{\prime }$ to the principal point $p_{0}$. For cameras with minimal distortion, it is often sufficient to determine $k_{1}$ and $k_{2}$ only. The extrinsic parameters, intrinsic parameters and radial distortion coefficients of the camera can be obtained using Zhang’s calibration method [32].

When the Risley prisms are placed in front of the camera, the imaging process of the system becomes highly complex. Due to the nonlinear characteristics of refraction, the pixel corresponding to the object point P will shift from $p^{\prime }$ to $p={\left[u,v\right]}^{T}$. In three-dimensional space, the refraction process of the light ray can be described by the following vector form [33]:

(4) $$r_{i+1}=\frac{n_{i}}{n_{i+1}}r_{i}+\frac{-n_{i}r_{i}^{T}n_{i+1}-\sqrt{n_{i}^{2}{\left(r_{i}^{T}n_{i+1}\right)}^{2}-\left(n_{i}^{2}-n_{i+1}^{2}\right)r_{i}^{T}r_{i}}}{n_{i+1}}$$

where i=0,1,2,3 and $n_{i+1}$ is the refractive index of the medium. When i is even, it corresponds to the refractive index of air, and when i is odd, it represents the refractive index of the Risley prisms. $n_{i+1}$ denotes the normal vector to the prism surface, and $r_{i+1}$ represents the direction of the light ray. The normal vector to the prism surface can be calculated using the following equation:

(5) $$n_{1}={\left[-\sin\alpha \sin\theta_{1},\sin\alpha \cos\theta_{1},\cos\alpha \right]}^{T}$$

(6) $$n_{2}={\left[\begin{array}{c} 0,0,1 \end{array}\right]}^{T}$$

(7) $$n_{3}={\left[0,0,1\right]}^{T}$$

(8) $$n_{4}={\left[\sin\alpha \sin\theta_{2},-\sin\alpha \cos\theta_{2},\cos\alpha \right]}^{T}$$

After lens distortion correction, the unit direction vector of the initial light ray corresponding to any pixel on the image can be expressed as:

(9) $$r_{0}=\frac{R^{-1}K^{-1}{\left[u,v,1\right]}^{T}}{\left|R^{-1}K^{-1}{\left[u,v,1\right]}^{T}\right|}$$

where $R^{-1}$ is the inverse matrix of the camera’s rotation matrix and $K^{-1}$ is the inverse matrix of the camera’s intrinsic matrix. According to the pinhole camera model, the direction of the initial light ray must pass through the optical center. Therefore, the starting point of the initial light ray is as follows:

(10) $$O=-R^{-1}T$$

To simplify the subsequent derivation process, we align the world coordinate system with the camera coordinate system, which means R becomes I (the 3 × 3 identity matrix) and $T={\left[0,0,0\right]}^{T}$. Consequently, the following formulas are derived in the camera coordinate system. Therefore, Equations (9) and (10) can be simplified as follows:

(11) $$r_{0}=\frac{K^{-1}{\left[u,v,1\right]}^{T}}{\left|K^{-1}{\left[u,v,1\right]}^{T}\right|}$$

(12) $$O={\left[\begin{array}{c} 0,0,0 \end{array}\right]}^{T}$$

In the process of ray tracing, let the intersection point of the ray $r_{i+1}$ and the prism surface be denoted as $a_{i+1}$, which can be calculated using the following formula:

(13) $$a_{i+1}=\frac{n_{i+1}^{T}\left(O_{i+1}-a_{i}\right)}{n_{i+1}^{T}r_{i}}r_{i}+a_{i}$$

where i=0,1,2,3 and $a_{0}=O$.

To provide a clearer and more structured understanding of the process described above, the procedure is summarized in Algorithm 1.

**Algorithm 1:** Ray Tracing Method Based on The Refractive Camera Model**Input:** Image point $p_{d}^{\prime }$, camera’s intrinsic matrix *K*, rotation angles $\theta_{1},\theta_{2}$ system parameters**Output:** Outgoing ray direction ***d***_out_1: Correct lens distortion using Equation (3) to obtain the ideal image point *p*′2: Compute initial ray direction ***d***_0_ using Equation (11)3: Set ray origin *O_c_* using Equation (12)4: **for** each refractive surface *i* = 1 → 4 **do**5:    Compute surface normal ***n**_i_* using Equations (5)–(8)6:    Compute intersection point using Equation (13)7:    Update ray direction using Snell’s law in vector form (Equation (4))8: **end for**9: **return** final outgoing ray direction ***d***_out_

### 2.3. Triangulation-Based 3D Reconstruction Model for Risley Prisms

**Figure 2:**
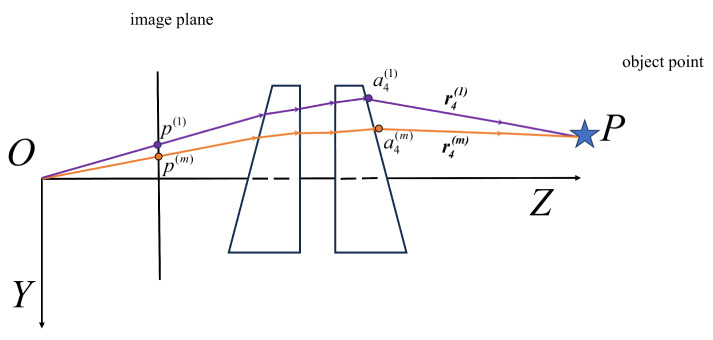
The principle of triangulation method for the Risley-prism 3D imaging system.

In general, 3D reconstruction based on images is essentially an ill-posed problem. Without prior information about the scene, a single two-dimensional image cannot provide sufficient constraints to uniquely determine the 3D structure of the target. According to multi-view geometry theory, at least two or more target images from different viewpoints are required to achieve 3D reconstruction. Here, the triangulation method is adopted to determine the 3D coordinates of objects. The specific processes are as follows:

First, we need to obtain a set of corresponding matching points $\left\{p^{\left(m\right)}|m=1,2,3\ldots ,M\right\}$ from the captured images, where M is the total number of images. Next, the corresponding set of direction vectors $\left\{r_{4}^{\left(m\right)}|m=1,2,3\ldots ,M\right\}$ can be calculated using Equations (4) and (11), and then $\left\{a_{4}^{\left(k\right)}|k=1,2,3\ldots ,m\right\}$ is determined using Equation (13). As shown in Figure 2, the object point P must lie on the line that starts from $a_{4}^{\left(m\right)}$ and extends in the direction of $r_{4}^{\left(m\right)}$. Therefore, the object point P can be expressed as:

(14) $$P=a_{4}^{\left(m\right)}+\mu r_{4}^{\left(m\right)}$$

where μ represents the scale factor, indicating the distance from $a_{4}^{\left(m\right)}$ to the point P along the direction $r_{4}^{\left(m\right)}$. In theory, these rays should intersect at the point P. However, due to factors such as parameter errors and noise, the M rays do not intersect exactly. Therefore, the point P is determined as the one that minimizes the sum of the distances from all rays, which is solved using the least squares method. To derive the analytical form of this solution, Equation (14) can be expanded to the three-dimensional coordinate form:

(15) $$\left[\begin{array}{c} x_{P}\\ y_{P}\\ z_{P} \end{array}\right]=\left[\begin{array}{c} x_{a_{4}}^{\left(m\right)}\\ y_{a_{4}}^{\left(m\right)}\\ z_{a_{4}}^{\left(m\right)} \end{array}\right]+\mu \left[\begin{array}{c} x_{r_{4}}^{\left(m\right)}\\ y_{r_{4}}^{\left(m\right)}\\ z_{r_{4}}^{\left(m\right)} \end{array}\right]$$

where ${\left[x_{P},y_{P},z_{P}\right]}^{T}$, ${\left[\begin{array}{c} x_{a_{4}}^{\left(m\right)},y_{a_{4}}^{\left(m\right)},z_{a_{4}}^{\left(m\right)} \end{array}\right]}^{T}$, and ${\left[\begin{array}{c} x_{r_{4}}^{\left(m\right)},y_{r_{4}}^{\left(m\right)},z_{r_{4}}^{\left(m\right)} \end{array}\right]}^{T}$ are the three-dimensional coordinates of P, $a_{4}^{\left(m\right)}$, and $r_{4}^{\left(m\right)}$, respectively. μ can be calculated using the following formula:

(16) $$\mu =\frac{\left(z_{P}-z_{a_{4}}^{\left(m\right)}\right)}{z_{r_{4}}^{\left(m\right)}}$$

Substituting Equation (16) into Equation (15), we obtain:

(17) $$\left[\begin{array}{c} z_{r_{4}}^{\left(m\right)}x_{P}-x_{r_{4}}^{\left(m\right)}z_{P}\\ z_{r_{4}}^{\left(m\right)}y_{P}-y_{r_{4}}^{\left(m\right)}z_{P} \end{array}\right]=\left[\begin{array}{c} z_{r_{4}}^{\left(m\right)}x_{a_{4}}^{\left(m\right)}-x_{r_{4}}^{\left(m\right)}z_{a_{4}}^{\left(m\right)}\\ z_{r_{4}}^{\left(m\right)}y_{a_{4}}^{m}-y_{r_{4}}^{\left(m\right)}z_{a_{4}}^{\left(m\right)} \end{array}\right]$$

When there are M corresponding points in the system, Equation (17) can be further expanded as follows:

(18) $$\left.\left[\begin{array}{c} z_{r_{4}}^{\left(1\right)}x_{P}-x_{r_{4}}^{\left(1\right)}z_{P}\\ z_{r_{4}}^{\left(1\right)}y_{P}-y_{r_{4}}^{\left(1\right)}z_{P}\\ \vdots \\ z_{r_{4}}^{\left(M\right)}x_{P}-x_{r_{4}}^{\left(M\right)}z_{P}\\ z_{r_{4}}^{\left(M\right)}x_{P}-x_{r_{4}}^{\left(M\right)}z_{P} \end{array}\right.\right]=\left[\begin{array}{c} z_{r_{4}}^{\left(1\right)}x_{a_{4}}^{\left(1\right)}-x_{r_{4}}^{\left(1\right)}z_{a_{4}}^{\left(1\right)}\\ z_{r_{4}}^{\left(1\right)}y_{a_{4}}^{\left(1\right)}-y_{r_{4}}^{\left(1\right)}z_{a_{4}}^{\left(1\right)}\\ \vdots \\ z_{r_{4}}^{\left(M\right)}x_{a_{4}}^{\left(M\right)}-x_{r_{4}}^{\left(M\right)}z_{a_{4}}^{\left(M\right)}\\ z_{r_{4}}^{\left(M\right)}y_{a_{4}}^{\left(M\right)}-y_{r_{4}}^{\left(M\right)}z_{a_{4}}^{\left(M\right)} \end{array}\right]$$

For the convenience of numerical calculation, Equation (18) can be rewritten in matrix form as:

(19) $$\left.\left[\begin{array}{ccc} z_{r_{4}}^{\left(1\right)} & 0 & x_{r_{4}}^{\left(1\right)}\\ 0 & z_{r_{4}}^{\left(1\right)} & y_{r_{4}}^{\left(1\right)}\\ \vdots & \vdots & \vdots \\ z_{r_{4}}^{\left(M\right)} & 0 & x_{r_{4}}^{\left(M\right)}\\ 0 & z_{r_{4}}^{\left(M\right)} & y_{r_{4}}^{\left(M\right)} \end{array}\right.\right]\left[\begin{array}{c} x_{P}\\ y_{P}\\ z_{P} \end{array}\right]=\left[\begin{array}{c} z_{r_{4}}^{\left(1\right)}x_{a_{4}}^{\left(1\right)}-x_{r_{4}}^{\left(1\right)}z_{a_{4}}^{\left(1\right)}\\ z_{r_{4}}^{\left(1\right)}y_{a_{4}}^{\left(1\right)}-y_{r_{4}}^{\left(1\right)}z_{a_{4}}^{\left(1\right)}\\ \vdots \\ z_{r_{4}}^{\left(M\right)}x_{a_{4}}^{\left(M\right)}-x_{r_{4}}^{\left(M\right)}z_{a_{4}}^{\left(M\right)}\\ z_{r_{4}}^{\left(M\right)}y_{a_{4}}^{\left(M\right)}-y_{r_{4}}^{\left(M\right)}z_{a_{4}}^{\left(M\right)} \end{array}\right]$$

In practice, the determination of the 3D coordinates of the point P needs to solve the overdetermined system of equations in Equation (19). The most commonly used methods for this problem include least squares and singular value decomposition (SVD). From the formulation in Equation (19), increasing the number of corresponding points M provides more geometric constraints for the least-squares solution, which improves the condition number of the system. This overdetermined system reduces the influence of random noise and matching errors by averaging residuals across multiple observations, thereby improving the robustness and accuracy of the 3D reconstruction results. Therefore, the proposed triangulation approach maintains both efficiency and robustness across a wide range of M values, as further supported by the multi-view geometry principle that additional views reduce the uncertainty of the reconstructed point.

## 3. The Impact of Errors on 3D Reconstruction

### 3.1. Forward Projection Calculation Method for Risley-Prism 3D Imaging System Based on Fermat’s Principle

The triangulation of the refractive cameras is essentially a reverse projection process, mapping 2D points on the image plane back to a line in 3D space. In contrast, forward projection is the process of mapping points in 3D space onto the image plane. However, unlike reverse projection, the forward projection calculation for refractive cameras is significantly complex, because it is difficult to determine which light ray originating from the object point will pass through the optical center of the camera after refraction. Agrawal et al. [34] pointed out that forward projection can be achieved by solving a quartic equation for one refractive interface, and a twelfth-degree equation should be solved for two parallel refractive interfaces. As the number of refractive interfaces increases, the computational complexity of forward projection grows exponentially.

**Figure 3:**
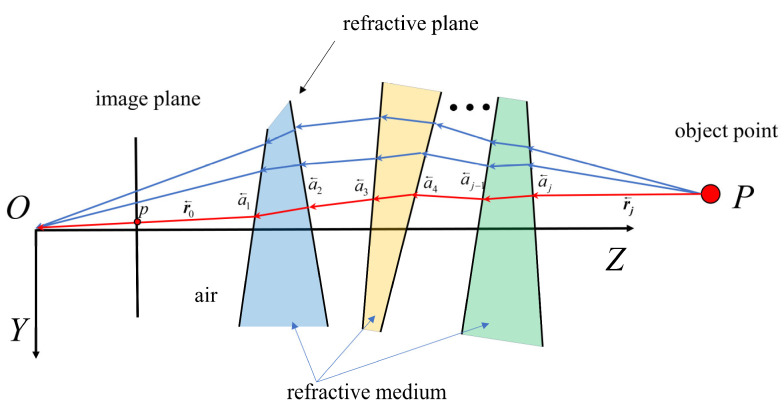
Schematic diagram of the forward projection of the refractive camera based on Fermat’s principle.

Therefore, it is necessary to propose a general forward projection calculation method that is applicable to any number of refractive interfaces. It is necessary to note that when there are J refractive interfaces, there is only one correct path for the ray from point P to point O, as illustrated in Figure 3. According to Fermat’s principle, light traveling through different media will take the path that minimizes the travel time to its destination. Thus, the forward projection problem can be transformed into an optimization problem to find the shortest optical path from point P to point O.

For an optimization problem, the first step is to define the objective function. Let the unit direction vector of the ray emitted from point P be denoted as ${\vec{r}}_{j}={\left[\eta ,\gamma ,\xi \right]}^{T}$. Since ${\vec{r}}_{j}$ is a unit vector, the variable η,γ,ξ must satisfy the following condition:

(20) $$\eta^{2}+\gamma^{2}+\xi^{2}=1$$

We can calculate ${\vec{r}}_{\left(j-1\right)}=(\eta ,\gamma ,\xi )$ based on Equation (4) and then calculate the intersection points ${\vec{a}}_{j}\left(\eta ,\gamma ,\xi \right),j\in [1,J]\cap \mathbb{Z}$ using Equation (13). Once these intersection points are obtained, the total optical path length of the ray can be calculated. Therefore, the problem can be formulated as follows:

(21) $$arg\;\underset{\eta ,\gamma ,\xi }{\min}\sum_{j=0}^{J}n_{j}\|{\vec{a}}_{j+1}\left(\eta ,\gamma ,\xi \right)-{\vec{a}}_{j}\left(\eta ,\gamma ,\xi \right)\|s.t.\mathrm{Eq}\left(20\right)$$

where ${\vec{a}}_{0}=O$, ${\vec{a}}_{j+1}=C$, and $n_{j}$ is the refractive index of the refractive medium $j_{th}$.

After calculating the initial direction of the ray emitted from point P using Equation (21), the pixel coordinates p of point P in the image plane can be determined using the pinhole camera model as follows:

(22) $$\lambda \bar{p}=K{\vec{a}}_{1}$$

When calculating the forward projection results for the Risley-prism 3D imaging system, we only need to convert the above parameters to those corresponding to the Risley prisms. For example, in the Risley-prism 3D imaging system, the number of refractive interfaces J is 4, and the normal vector of the refractive interface $n_{j}$ can be calculated using Equations (5)–(8). But a negative sign needs to be added before the equation, i.e., $n_{j}=-n_{j}$.

The forward projection process, formulated as an optimization problem based on Fermat’s principle, is detailed in Algorithm 2.

**Algorithm 2**: Forward Projection Based on Fermat’s Principle**Input:** 3D point *P*, prism rotation angles $\theta_{1},\theta_{2}$, system parameters, max_iter, tolerance *ε***Output:** Pixel coordinates *p*1: Initialize ray direction $d_{0}$ from *P*2: **for** each candidate direction ***d* do**3:    Trace ray through all refractive surfaces using Equations (4) and (13)4:    Compute total optical path length *L*(***d***) using Equation (21)5: **end for**6: Select the direction d^*^ that minimizes *L*(***d***)7: **while** not converged **do**8:    Refine ***d*** using gradient-based optimization with constraint ‖d‖=19:    Update ray path and recompute *L*(***d***)10:    **if** $\left|L_{\mathrm{new}}-L_{\mathrm{old}}\right|<\varepsilon$ or iteration count > max_iter **then**11:     break12:    **end if**13: **end while**14: Project final ray onto image plane using Equation (22) to obtain pixel coordinates *p*15: **return**
*p*

### 3.2. Error Analysis of the Risley-Prism 3D Imaging System

#### 3.2.1. Sources of Error

**Figure 4:**
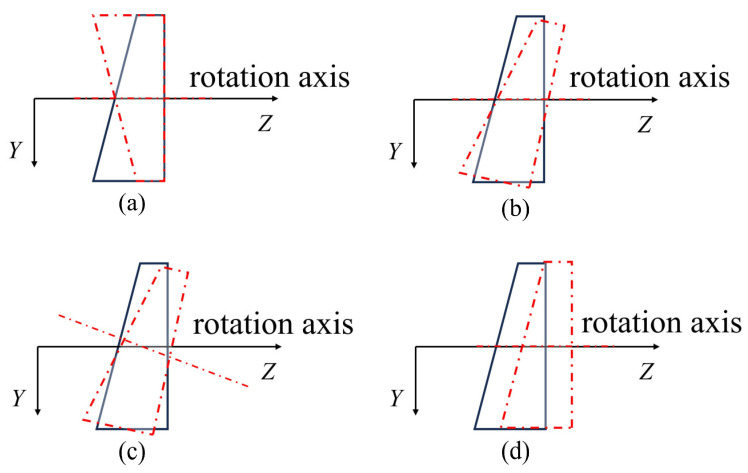
Schematic diagram of errors in the Risley-prism 3D imaging system. The solid black line represents the ideal state of the system, while the red dotted line represents the actual state: (**a**) prism orientation error, (**b**) prism tilt error, (**c**) bearing tilt error, (**d**) prism translation error.

In the modeling process described, it is assumed that the Risley-prism 3D imaging system is in an ideal state, which means that there are no errors. However, in practical applications, the Risley-prism 3D imaging system is subject to various errors, which can generally be categorized into two main types: mechanical installation errors and manufacturing errors. Mechanical installation errors primarily include the following four types: prism orientation errors, prism tilt errors, bearing tilt errors, and prism translation errors, as shown in Figure 4 [35,36]. The above error parameters are summarized in Table 1. Compared to mechanical installation errors, manufacturing errors are considered negligible due to high-precision manufacturing processes.

#### 3.2.2. Error Model

To characterize the influence of these errors, we use rotation matrices $R_{\theta s}$, $R_{ps}$ and $R_{bs}$, respectively, to describe prism orientation errors, prism tilt errors, and bearing tilt errors, where the subscript s indicates the prism number (either 1 or 2). According to Rodrigues’ rotation formula, the rotation matrix R rotating by β degrees around any axis a can be expressed as [37]:

(23) $$R=ℜ\left(a,\beta \right)=D+\left(I-D\right)\cos\beta +G\sin\beta$$

where I is the 3 × 3 identity matrix and D and G are matrices related to the rotation axis $a={\left[x_{a},y_{a},z_{a}\right]}^{T}$, which can be expressed as follows:

(24) $$D=\left[\begin{array}{ccc} x_{a}^{2} & x_{a}y_{a} & x_{a}z_{a}\\ x_{a}y_{a} & y_{a}^{2} & y_{a}z_{a}\\ x_{a}z_{a} & y_{a}z_{a} & x_{a}^{2} \end{array}\right],G=\left[\begin{array}{ccc} 0 & -z_{a} & y_{a}\\ z_{a} & 0 & -x_{a}\\ y_{a} & x_{a} & 0 \end{array}\right]$$

Based on the above equations, the prism tilt error rotation matrix $R_{ps}$ and the bearing tilt error rotation matrix $R_{bs}$ can be expressed as follows:

(25) $$R_{ps}=ℜ\left(a_{ps},\beta_{ps}\right)=ℜ\left(\left[\cos\Phi_{ps},\sin\Phi_{ps},0\right],\beta_{ps}\right)$$

(26) $$R_{bs}=ℜ\left(a_{bs},\beta_{bs}\right)=ℜ\left(\left[\cos\Phi_{bs},\sin\Phi_{bs},0\right],\beta_{bs}\right)$$

When there are bearing tilt errors, the prism rotation axis will shift from the *Z*-axis to the axis $a_{\theta s}$, which can be calculated using the following equation:

(27) $$a_{\theta s}=R_{bs}{\left[0,0,1\right]}^{T}$$

Thus, the prism orientation error matrix $R_{\theta s}$ will rotate around the $a_{\theta s}$ axis by $\Delta \theta_{s}$ degrees:

(28) $$R_{\theta s}=ℜ\left(\left[\sin\Phi_{bs}\sin\beta_{bs},-\cos\Phi_{bs}\sin\beta_{bs},\cos\beta_{bs}\right],\theta_{s}+\Delta \theta_{s}\right)$$

In addition to the rotational errors mentioned above, there is also the prism translation error $t_{ps}={\left[t_{xs},t_{ys},t_{zs}\right]}^{T}$. From the analysis of error types in the Risley-prism system, it can be concluded that the prism orientation errors, tilt errors, and translation errors collectively describe the rigid body transformation of the prism in three-dimensional space, while the bearing tilt error characterizes the transformation of the prism’s rotation axis. Therefore, the errors in the Risley-prism system can be characterized by the above equations.

When considering the impact of actual errors, the equations derived under ideal conditions are no longer applicable. The normal vector of the prism surface will change from Equations (5)–(8) to the following form:

(29) $$n_{1}^{e}=R_{\theta 1}R_{p1}n_{1}$$

(30) $$n_{2}^{e}=R_{\theta 2}R_{p2}n_{2}$$

(31) $$n_{3}^{e}=R_{\theta 3}R_{p3}n_{3}$$

(32) $$n_{4}^{e}=R_{\theta 4}R_{p4}n_{4}$$

Substituting the new prism surface normal vector $n_{i+1}^{e}$ into Equation (4), the actual ray direction vector can be calculated when errors are taken into account:

(33) $$r_{i+1}^{e}=\frac{n_{i}}{n_{i+1}}r_{i}^{e}+\frac{-n_{i}{\left(r_{i}^{e}\right)}^{T}n_{i+1}^{e}-\sqrt{n_{i}^{2}{\left({\left(r_{i}^{e}\right)}^{T}n_{i+1}^{e}\right)}^{2}-(n_{i}^{2}-{n_{i+1}}^{2}){\left(r_{i}^{e}\right)}^{T}(r_{i}^{e})}}{n_{i+1}}$$

In addition, owing to the introduction of the prism translation error, the position of the intersection point between the light ray and the prism surface would change. By incorporating the surface normal vector with prism errors and the translation error into Equation (13):

(34) $$P_{i+1}^{e}=\frac{{\left(n_{i+1}^{e}\right)}^{T}\left(O_{i+1}^{e}-P_{i}^{e}\right)}{{\left(n_{i+1}^{e}\right)}^{T}r_{i}^{e}}r_{i}^{e}+P_{i}^{e}$$

(35) $$O_{i+1}^{e}=\left\{\begin{array}{ll} O_{i+1}+t_{ps}-P_{i}^{e} & \mathrm{when}\;i=0,2\\ O_{i+1}+t_{ps}+d_{2}n_{i+1}^{e}-P_{i}^{e} & \mathrm{when}\;i=1,3 \end{array}\right.$$

where $d_{2}$ represents the thickness of the prism center.

### 3.3. Evaluation of Reconstruction Errors

In order to comprehensively evaluate 3D reconstruction accuracy, we employ the Normalized Root Mean Square Error (NRMSE) and Standard Deviation (SD) of 3D points as the primary evaluation metrics.

The Normalized Root Mean Square Error is one of the commonly used metrics in 3D reconstruction to quantify the difference between the reconstruction results and the true values. It provides a substantial indication of the precision of the reconstruction results and is defined as follows [38]:

(36) $$E_{q}=\sqrt{\|{\tilde{A}}^{\left(q\right)}-A^{\left(q\right)}{\|}^{2}}$$

(37) $$RMSE=\sqrt{\frac{1}{Q}\sum_{q=1}^{Q}E_{q}^{2}}$$

(38) $$NRMSE=\frac{RMSE}{\left\|A_{\max}-A_{\min}\right\|}$$

where $\tilde{A}$ and A represent the coordinates of the reconstructed 3D point and the actual 3D point, respectively, and also the measured value and the reference value; Q is the total number of reconstructed points; q is the index of the reconstructed point; and $\left\|A_{\max}-A_{\min}\right\|$ is the range of the actual 3D points. The NRMSE is usually expressed as a percentage. The smaller the value of the NRMSE, the higher the accuracy of the reconstruction result.

The Standard Deviation (SD), which describes the error distribution of reconstructed 3D points, is also one of the commonly used indicators in 3D reconstruction. The expression for SD is as follows:

(39) $$SD=\sqrt{\frac{1}{Q}\sum_{q=1}^{Q}{\left(E_{q}-\bar{E}\right)}^{2}}$$

where $\bar{E}=\frac{1}{Q}\sum_{q=1}^{Q}E_{q}$.

### 3.4. Simulation Analysis of the Influence of Errors on the Imaging Accuracy

#### 3.4.1. System Parameters

To analyze the impact of errors on the reconstruction results, simulation experiments were conducted based on the Risley-prism 3D imaging system. The parameters of the Risley-prism 3D imaging system are as follows: $\alpha_{1}=\alpha_{2}=\alpha =11.35{}^{\circ}$, $n_{1}=n_{2}=n=1.515$, $d_{1}=26.99$ mm, $d_{2}=d_{4}=5.51$ mm, $d_{3}=2$ mm, ρ=5.51 mm. The focal length of the camera is f=12 mm, with an image size of 2048 × 3072 pixels, and the pixel size is $d_{x}=d_{y}=2.4$ µm. A checkerboard pattern was used as the target for reconstruction, with a distance of 50 mm between adjacent corners. The checkerboard plane was positioned perpendicular to the camera’s optical axis and 2500 mm away from the camera’s optical center, with a total of 9 × 19 corner points. Based on the above parameters and the forward projection calculation method, simulation data were generated for error analysis.

#### 3.4.2. Analysis of the Impact of Angular Errors

**Figure 5:**
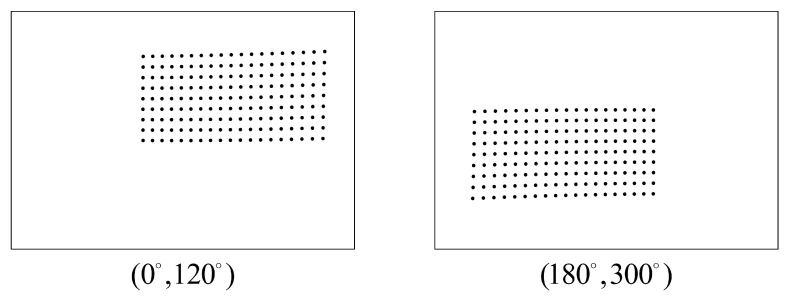
Schematic diagram of pixel coordinates in the camera’s checkerboard pattern. The left image illustrates the simulation result when the prism orientation angles are (0°,120°), while the right image shows the simulation result for prism orientation angles of (180°,300°). The distance between the checkerboard and the camera is 2500 mm.

Angular errors include prism orientation errors, prism tilt errors, and bearing tilt errors. Simulation experiments were conducted to reconstruct the checkerboard corner points using two views, with prism orientations $\left(\theta_{1},\theta_{2}\right)$ set to (0°,120°) and (180°,300°), respectively. The pixel coordinates of the checkerboard corners are shown in Figure 5.

**Figure 6:**
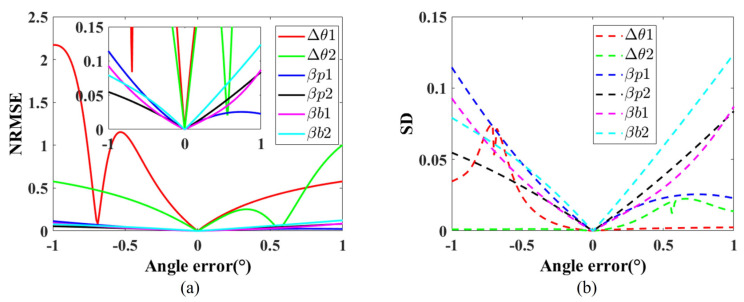
The effect of angular errors on the 3D reconstruction results, where $\Delta \theta_{1}$ and $\Delta \theta_{2}$ represent the prism orientation errors, $\beta_{p1}$ and $\beta_{p2}$ represent the prism tilt angles, and $\beta_{b1}$ and $\beta_{b2}$ denote the bearing tilt errors. The tilt axis for both is denoted by $a={\left[1,0,0\right]}^{T}$. (**a**) The relationship between angular errors and NRMSE. (**b**) The relationship between angular errors and SD.

The simulation results are shown in Figure 6. As can be seen in Figure 6, the impact of prism orientation errors on the reconstruction results is significantly greater than that of prism tilt errors and bearing tilt errors. To better visualize the impact of prism angle errors on reconstruction results, Figure 6a also presents a magnified view of the NRMSE axis, limited to the range [0, 0.15] while maintaining the full angular error range. This detailed perspective reveals that although prism tilt errors and bearing tilt errors do have a measurable impact on reconstruction accuracy, their effect is orders of magnitude smaller than that of prism orientation errors, further confirming that prism orientation errors are the primary factor limiting reconstruction accuracy in the Risley-prism imaging system.

**Figure 7:**
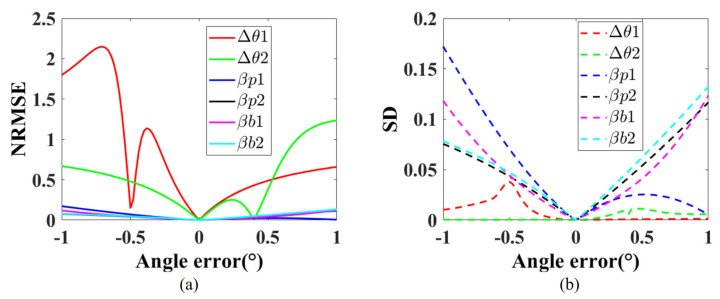
The effect of angular errors on the 3D reconstruction results, when the object points are at a distance of 3500 mm from the camera. (**a**) The relationship between angular errors and NRMSE. (**b**) The relationship between angular errors and SD.

To further consider the impact of angular errors at different distances, we conducted another experiment by moving the checkerboard 1000 mm further away, resulting in a distance of 3500 mm between the checkerboard and the camera. The results are shown in Figure 7. As shown in Figure 7, the curves exhibit analogous behavior to those in Figure 6, but they are not identical due to the increased distance. This also demonstrates that the angular errors at different distances have a similar effect on the reconstruction results.

#### 3.4.3. Analysis of the Influence of Translation Error

**Figure 8:**
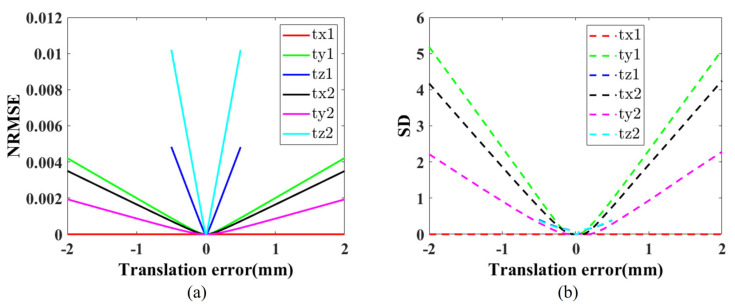
The effect of translation errors on the 3D reconstruction results, where $t_{p1,2}={\left[t_{x1,2},t_{y1,2},t_{z1,2}\right]}^{T}$ represents the translation errors of prisms 1 and 2. (**a**) The relationship between translation errors and NRMSE. (**b**) The relationship between translation errors and SD.

Translation errors along the *X*, *Y*, and *Z* directions were analyzed, and the results are shown in Figure 8. Results indicate that *Z*-direction translation errors have the greatest impact on NRMSE due to their influence on all prism planes. And because of the normal vector of the prism surface, the translation error of prism 1 in the *X* direction has no effect on the NRMSE when the plane’s normal vector is perpendicular to a certain axis.

#### 3.4.4. Analysis of Overall Orientation Angle and Noise Impact

**Figure 9:**
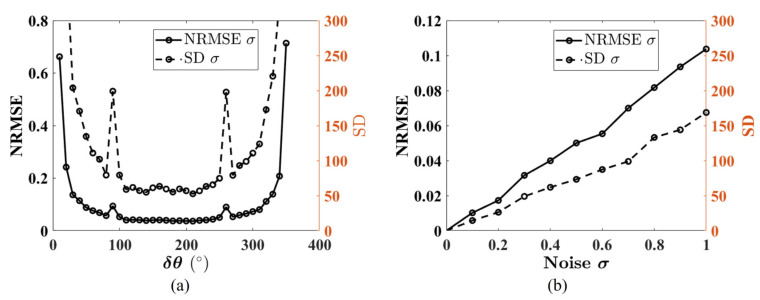
The effect of noise and overall orientation on the 3D reconstruction results. (**a**) The relationship between overall orientation δθ and NRMSE and SD. (**b**) The relationship between noise σ and NRMSE and SD.

In practical applications, matching between points is often subjected to various factors, leading to a certain degree of error in the matching results. To simulate the effect of image matching errors and analyze the influence of the choice of overall orientation angle that was applied to both prisms, as well as noise, on reconstruction accuracy, Gaussian noise with zero mean is added to the pixel coordinates of the checkerboard corners. The noise intensity is controlled by its standard deviation σ, tested over the range [0, 1]. The simulation results are depicted in Figure 9.

Figure 9a illustrates the relationship between the overall orientation angle δθ and both the NRMSE and SD when imaging noise, σ=0.5. As shown in Figure 9a, the NRMSE is nearly symmetric about δθ=180°, reaching its minimum value around δθ=180°. This phenomenon is similar to the effect of baseline length on reconstruction accuracy for stereo vision, and the δθ is analogous to the baseline in stereo vision [21].

For the 3D reconstruction based on multiple images, the accuracy of image matching also has an impact on the reconstruction accuracy. The simulation of the effect of image mismatches on the reconstruction accuracy is carried out, and Gaussian noise is used to model mismatches. Figure 9b shows the relationship between noise level σ and both NRMSE and SD, with σ in the range [0, 1]. The results show that as σ increases, both NRMSE and SD exhibit a monotonically increasing trend. This phenomenon is consistent with theoretical expectations, indicating that the enhancement of matching noise significantly reduces the positioning accuracy and stability of the system.

#### 3.4.5. Summary of Error Impact

Based on the above error analyses, the influence of errors on 3D reconstruction can be ranked as follows: orientation errors of Prism 1 > orientation errors of Prism 2 ≫ tilt errors of Prism 1 ≈ tilt errors of Prism 2 ≈ Bearing tilt errors of Prism 1 ≈ Bearing tilt errors of Prism 2 ≫ *Z* direction translation errors of Prism 2 > *Z* direction translation errors of Prism 1 > *X*, *Y* direction translation errors of Prism 1 ≈ *X*, *Y* direction translation errors of Prism 2.

It should be noted that this conclusion is reached under prism orientation angles of (180°,300°). Relevant simulation experiments were also conducted under other prism orientation angles. Although there are slight numerical differences, the overall trend remains consistent. In addition, the simulation results indicate that it is advisable to minimize the impact of noise while keeping the angular difference between the two sets of prisms at around δθ=180°.

## 4. Calibration Method of Orientation Errors

As the above research shows, the Risley-prism 3D imaging system involves a total of 16 error sources. It is impractical to carry out the optimization correction of all error variables. Based on the above error analyses, it can be concluded that the primary factor affecting 3D reconstruction accuracy is the prism orientation error. Therefore, the calibration method of the prism orientation errors is proposed to enhance the precision of the 3D reconstruction.

A checkerboard with a total of 6 × 9 inner corners and a square size of 25 mm is used as the calibration target in the calibration process. During calibration, two parallel views are obtained by moving the camera horizontally without prisms, and the 3D coordinates of the checkerboard corner points are calculated based on the principle of binocular stereo vision. It should be noted that the calibration is performed once per experimental group and not averaged across groups. According to the error model in Section 3.2.2, the intersection point $P^{e}\left(\Delta \theta_{1},\Delta \theta_{2}\right)$ between the final outgoing ray and the checkerboard plane can be calculated. It is important to note that, since only the prism orientation error is considered, the matrices $R_{ps}$ and $R_{bs}$ in Equations (25) and (26) should be replaced with identity matrices.

Ideally, if the system parameters are accurate and the corner points are sufficiently precise, the corner point coordinates P calculated by binocular stereo vision should be the same as $P^{e}\left(\Delta \theta_{1},\Delta \theta_{2}\right)$. But in reality, the system parameters cannot be completely accurate, so there are differences between P and $P^{e}\left(\Delta \theta_{1},\Delta \theta_{2}\right)$. Therefore, the orientation errors $\left(\Delta \theta_{1},\Delta \theta_{2}\right)$ can be obtained by minimizing the objective function, which is defined as the Euclidean distance between the point P and the point $P^{e}\left(\Delta \theta_{1},\Delta \theta_{2}\right)$, and the function can be expressed as follows:

(40) $$arg\underset{\Delta \theta_{1},\Delta \theta_{2}}{\min}\sum_{v=1}^{V}{\left\|P_{v}^{e}\left(\Delta \theta_{1},\Delta \theta_{2}\right)-P_{v}\right\|}^{2}$$

where V represents the total number of checkerboard corner points and the initial values of the orientation errors $\left(\Delta \theta_{1},\Delta \theta_{2}\right)$ can be set to (0°,0°). Equation (40) can be optimized using the Levenberg–Marquardt algorithm [39] or the Particle Swarm Optimization (PSO) algorithm [40].

It should be noted that the proposed calibration method focuses exclusively on prism orientation errors, as they are identified as the dominant factor affecting reconstruction accuracy under typical operating conditions. However, in scenarios involving significant mechanical misalignments or manufacturing defects, other error sources—such as translation or tilt errors—may become non-negligible. In such cases, the optimization framework can be naturally extended to incorporate additional error parameters, albeit at the cost of increased computational complexity and potential identifiability issues. Therefore, the applicability of the current method is primarily justified under the assumption that orientation errors dominate, which is consistent with both the simulation and experimental observations in this study.

## 5. Experimental Validation

To validate the effectiveness of the proposed refractive camera model, simulation analyses, and the calibration method for orientation errors, a series of 3D reconstruction experiments were conducted using the Risley-prism imaging system. This section details the experimental setup, procedures, and comparative results to demonstrate the performance of the proposed approach.

### 5.1. Experimental Setup

**Figure 10:**
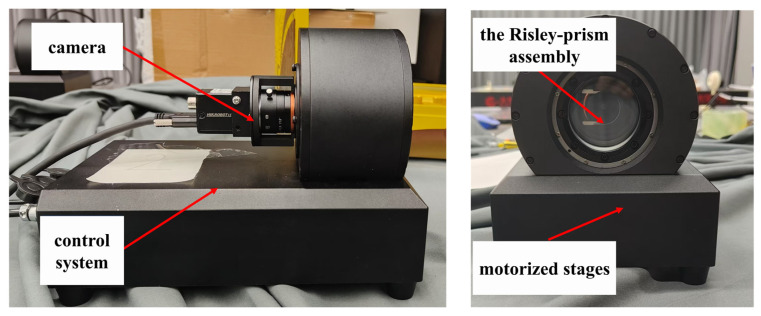
The experimental system diagram.

The experimental system was constructed in accordance with the simulation parameters described in Section 3.4.1, as shown in Figure 10. The core components included a Hikvision monocular camera (Hangzhou Hikrobot Co., Ltd., Hangzhou, China) equipped with a 12 mm fixed-focal-length lens. The camera’s intrinsic parameters were consistent with those used in simulations: a focal length f=12 mm, an image resolution of 2048 × 3072 pixels, and a pixel size of $d_{x}=d_{y}=2.4$ µm. The Risley-prism assembly, comprising two identical prisms with a wedge angle α=11.35° and refractive index n=1.515, was mounted in front of the camera. The prisms were independently rotatable via high-precision motorized stages. A standard checkerboard pattern with 6 × 9 inner corners and a square side length of 25 mm served as the calibration and reconstruction target. The checkerboard was positioned approximately 1892 mm from the camera’s optical center, with its plane carefully aligned to be perpendicular to the optical axis. All experiments were conducted under stable LED illumination to ensure consistent image quality.

### 5.2. Experimental Procedure

The experimental procedure consisted of the following key steps:

(1) System Initialization and Image Acquisition: The initial prism orientation was set to $\left(\theta_{1},\theta_{2})=(0{}^{\circ},120{}^{\circ}\right)$. A sequence of seven images was acquired by incrementally rotating both prisms simultaneously by 30° for each subsequent capture. The resulting orientation sets were: (0°,120°), (30°,150°), (60°,180°), (90°,210°), (120°,240°), (150°,270°) and (180°,300°)
(2) Orientation Error Calibration: The initial image (0°,120°) was paired with each of the subsequent six images to form six experimental groups. For each group, the prism orientation errors $\left(\Delta \theta_{1},\Delta \theta_{2}\right)$ were calibrated using the optimization-based method outlined in Section 4. The objective function (Equation (40)), minimizing the Euclidean distance between the 3D points obtained from binocular stereo vision (serving as the ground truth) and the points projected using the refractive model, was solved via the Levenberg–Marquardt algorithm.
(3) 3D Reconstruction: Following the calibration of orientation errors for each image pair, the 3D coordinates of the checkerboard corners were reconstructed using the triangulation method for refractive cameras detailed in Section 2.3.
(4) Performance Evaluation: The accuracy of the 3D reconstruction was quantitatively evaluated using the Normalized Root Mean Square Error (NRMSE) and Standard Deviation (SD), as defined in Section 3.3.

### 5.3. Results and Discussion

The calibration results for the prism orientation errors across the different image pairs are summarized in Table 2. As depicted in Table 2, the actual calibration results of the prism’s orientation have fluctuations, indicating that there are interference factors in the system that cannot be ignored, such as image mismatch and errors of the control system.

**Figure 11:**
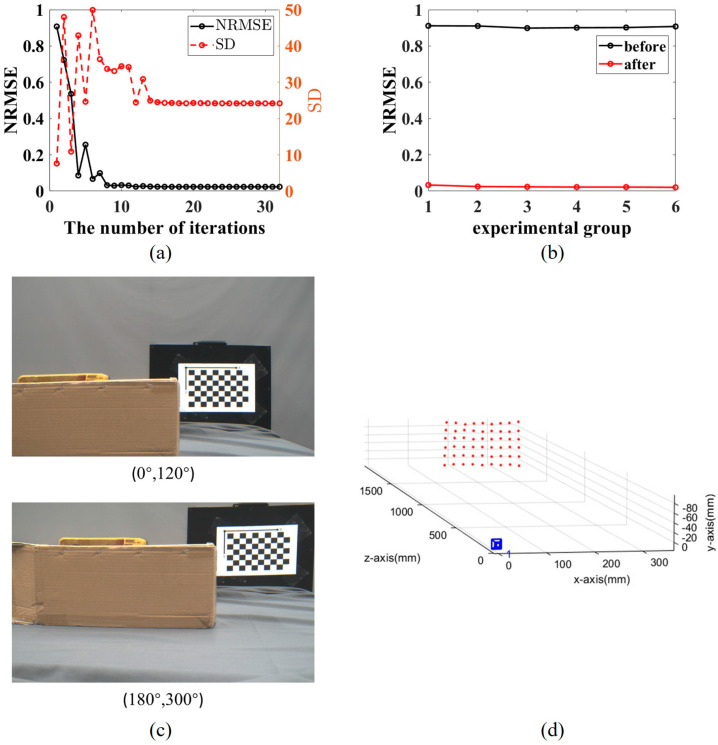
The experimental results for the Risley-prism 3D imaging system. (**a**) The relationship between the number of iterations and the NRMSE and SD during the prism orientation error calibration in the 6th group of experiments. (**b**) The changes in NRMSE for 6 sets of experiments before and after calibration. (**c**) Sixth set of experimental images captured. (**d**) 3D reconstruction results of the checkerboard corresponding to the sixth group of experiments.

The reconstruction performance is illustrated in Figure 11. Figure 11a depicts the convergence behavior of the NRMSE and SD during the iterative calibration process for Group 6. Prior to calibration, the initial NRMSE and SD were 0.9076 and 7.6147, respectively, indicating significant reconstruction errors primarily due to uncalibrated orientation errors. As the number of iterations increased, the NRMSE decreased consistently, while the SD initially increased slightly before decreasing, with both metrics stabilizing after approximately 32 iterations. The final NRMSE was reduced to 0.0207, demonstrating a substantial improvement in accuracy.

Figure 11b compares the NRMSE for all six experimental groups before and after the proposed calibration. A significant reduction in NRMSE is observed across all groups post-calibration, validating the effectiveness of the orientation error calibration method in enhancing 3D reconstruction quality. The captured target images for Group 6 and the corresponding 3D reconstruction results are shown in Figure 11c and Figure 11d, respectively, providing visual confirmation of the system’s capability.

The experimental results align well with the simulation analyses presented in Section 3, confirming that prism orientation errors are the dominant factor affecting reconstruction accuracy in the Risley-prism system. The proposed calibration and reconstruction framework successfully mitigates these errors, leading to a remarkable improvement in performance, as evidenced by the NRMSE reduction from 0.9076 to 0.0207.

## 6. Conclusions

In conclusion, this paper proposes a ray-tracing model for refractive cameras by combining the pinhole camera model with the vector form of Snell’s law. In addition, a forward projection method for refractive cameras based on Fermat’s principle is established, which offers a method from the object space to the image domain and is theoretically applicable to imaging systems with any number of refractive surfaces. In order to evaluate the impact of various errors on 3D reconstruction, this paper establishes an error analysis framework and carries out systematic simulation for quantitative evaluation based on the forward projection method. The simulation results show that prism orientation errors have the greatest impact on the accuracy of the 3D reconstruction among various error sources. Given the unique characteristics of the Risley-prism 3D imaging system, this paper innovatively proposes a triangulation method combining error calibration based on the optimization iteration to achieve 3D reconstruction of the target. The experimental results show that this calibration method can effectively improve the accuracy of the system’s 3D reconstruction, with the NRMSE index decreasing from the initial 0.9076 to 0.0207.

Despite these promising results, several limitations should be acknowledged:

(1) The current experimental validation is conducted under the controlled lab conditions using a static checkerboard target with a narrow field of view, which may not represent real-world complexity.
(2) Although 16 error sources exist in the system, only orientation errors are calibrated—other error sources may become significant under extreme conditions. Furthermore, imperfections in the prism material (e.g., manufacturing tolerances or material inhomogeneity) are not considered in this study, which may affect the imaging performance.

Therefore, future work will include: (1) validating the system’s performance in more complex and dynamic real-world scenarios, such as outdoor environments or applications requiring the reconstruction of non-cooperative targets; (2) further analyzing the applicability conditions of the current error calibration and extending it to incorporate additional error terms when necessary, as well as investigating the effects of prism material properties and manufacturing quality on reconstruction accuracy; (3) comparing the proposed method with other calibration methods—such as those based on homography or bundle adjustment—to provide further insight into the relative strengths and limitations of the proposed approach; and (4) exploring integration with deep learning-based 3D reconstruction methods using the calibrated multi-view images from the Risley-prism system as input for techniques like 3D Gaussian Splatting.

## Figures and Tables

**Table 1:** Summary of parameter errors in the Risley-prism 3D imaging system.

System Parameters	Error
Prism 1 orientation $\theta_{1}$	$\Delta \theta_{1}$
Prism 2 orientation $\theta_{2}$	$\Delta \theta_{2}$
Prism 1 tilt axis $a_{p1}$	$\Phi_{p1}$
Prism 2 tilt axis $a_{p2}$	$\Phi_{p2}$
Prism 1 tilt angle $\beta_{p1}$	$\beta_{p1}$
Prism 2 tilt angle $\beta_{p2}$	$\beta_{p2}$
Bearing 1 tilt axis $a_{b1}$	$\Phi_{b1}$
Bearing 2 tilt axis $a_{b2}$	$\Phi_{b2}$
Bearing 1 tilt angle $\beta_{b1}$	$\beta_{b1}$
Bearing 2 tilt angle $\beta_{b2}$	$\beta_{b2}$
Prism 1 translation $t_{p1}$	$t_{p1}$
Prism 2 translation $t_{p2}$	$t_{p2}$

**Table 2:** Optimization-based calibration results of prism orientation errors for different image groups.

NO.	$\Delta \theta_{1}$/°	$\Delta \theta_{2}$/°
1	4.91	−9.09
2	4.82	−8.77
3	5.04	−7.96
4	3.53	−7.59
5	3.67	−8.03
6	3.80	−8.09
7	4.43	−8.87

## Data Availability

The origin contributions presented in this study are included in the article. Further inquiries can be available on request from the corresponding author.
